# Intraoperative OCT bei Netzhautablösung mit Makulabeteiligung

**DOI:** 10.1007/s00347-020-01238-8

**Published:** 2020-10-06

**Authors:** V. Degenhardt, R. Khoramnia, J. Storr, C. S. Mayer

**Affiliations:** 1 Klink und Poliklinik für Augenheilkunde, Universität Heidelberg, Im Neuenheimer Feld 400, 69120 Heidelberg, Deutschland; 2 Klinik und Poliklinik für Augenheilkunde, Klinikum rechts der Isar, Technische Universität München, Ismaningerstraße 22, 81675 München, Deutschland

**Keywords:** Endotamponaden in der Bildgebung, Makulachirurgie, Netzhautfalten, Amotio retinae, Imaging of endotamponades, Macular surgery, Retinal folds, Retinal detachment

## Abstract

**Hintergrund:**

Seit wenigen Jahren ermöglicht die intraoperative optische Kohärenztomographie (iOCT) die Darstellung retinaler Strukturen im Operationsmikroskop.

**Ziel:**

Das Ziel dieser Studie ist die Analyse des Verhaltens der Netzhaut und die morphologische Befundung während Operationen bei Ablatio retinae mit Makulabeteiligung.

**Methoden:**

Retrospektive Analyse von 41 konsekutiven Augen mit Makula-off-Ablatio, welche mittels Pars-plana-Vitrektomie (ppV) unter Verwendung von iOCT behandelt wurden, durch qualitative Beurteilung der Netzhautmorphologie zu definierten Zeitpunkten im OP-Ablauf.

**Ergebnisse:**

In 63 % der Fälle gelang die iOCT-Darstellung der abgehobenen Makula, in den anderen Fällen überstieg die Netzhautabhebung die maximale Scantiefe der iOCT. Unter Perfluorodecalin zeigten 53,7 % der Augen noch subretinale Flüssigkeit, und 22 % der Augen zeigten eine Falte der äußeren Netzhautschichten. In 61 % der Augen konnte persistierende subretinale Flüssigkeit unter der endgültigen Tamponade festgestellt werden. Die subretinale Flüssigkeit konnte intraoperativ in dieser Menge mit bloßem Auge nicht festgestellt werden. In einem Fall konnte intraoperativ ein Makulaformen neu erkannt werden, in 3 Fällen zeigte sich zum Operationszeitpunkt eine wieder anliegende Makula.

**Diskussion:**

Die Informationen der iOCT führen eher selten zu einer Änderung oder Erweiterung des Eingriffs. Sie liefert jedoch in Echtzeit neue Informationen über intra- und subretinale Flüssigkeitsverteilung, die nicht immer der klinischen Einschätzung entsprechen. Die Bedeutung von persistierender subretinaler Flüssigkeit und die Falten der äußeren Netzhautschichten am Ende der Operation sind noch unklar. Sie könnten möglicherweise als prognostischer Faktor für das postoperative Outcome dienen.

## Abstract (en)

**Background:**

Intraoperative optical coherence tomography (iOCT) has recently been introduced for the visualization of retinal structures within the operating microscope.

**Objective:**

The aim of this study was to analyze the behavior of the retina and morphological features during surgery for retinal detachment with macular involvement.

**Methods:**

A retrospective analysis of 41 consecutive eyes with macula-off retinal detachment, which were treated with pars plana vitrectomy (PPV) using iOCT. A qualitative analysis of morphological features of the retina at defined moments during surgery was carried out.

**Results:**

The visualization of macular detachment with iOCT was successful in 63% of cases and in the other cases the height of retinal detachment surpassed the maximum depth of the scan by iOCT. With perfluorodecalin 53.7% of eyes showed subretinal fluid and 22% of eyes showed a wave-like configuration of the outer retinal layers. In 61% of the eyes persisting subretinal fluid could be detected under the final tamponade. The amount of subretinal fluid could not be detected intraoperatively with the naked eye. In one case a macular hole could be newly identified intraoperatively and in three cases macular detachment could not be found at the start of surgery, although a detachment had been expected.

**Conclusion:**

The use of iOCT rarely leads to changes or extension of the intervention; however, it provides real-time information on intraretinal and subretinal fluid, which is sometimes in contrast to the clinical assessment. The relevance of persistent subretinal fluid and folds of the outer retinal layers after surgery remains unclear. It can be speculated whether this could serve as a prognostic factor for the postoperative outcome.

Während die prä- und postoperative Untersuchung der Makula mittels optischer Kohärenztomographie (OCT) schon seit Jahren in weiten Bereichen zur Diagnostik, operativen Indikationsstellung und postoperativen Verlaufskontrolle bei vitreoretinalen Eingriffen gehört [3, 8, 9], ist die intraoperative Anwendung dieser Methode erst seit jüngerer Zeit möglich [2, 5, 6, 10, 16, 17] Mithilfe der intraoperativen optischen Kohärenztomographie (iOCT) können Schnittbilder retinaler Strukturen in Echtzeit in das Okular eines Operationsmikroskops eingeblendet werden. Wo bisher nur ein En-face-Bild zur Verfügung stand, wird dieses durch die iOCT um eine Vielzahl von Informationen durch die vertikalen Schnittbilder ergänzt. Erkenntnisse, welche durch eine iOCT während einer laufenden Operation gewonnen werden, können die sofortige Entscheidung über chirurgische Strategien beeinflussen [11–15]. Bisher ist nur wenig über das intraoperative Verhalten der Retina im Rahmen von netzhautchirurgischen Manipulationen bekannt. Ziel dieser Arbeit ist die Analyse von iOCT-Aufnahmen der zentralen Netzhaut, welche bei der operativen Versorgung von Netzhautablösungen mit Makulabeteiligung entstanden sind. Insbesondere sollten dabei die morphologischen Gegebenheiten der Makula im Hinblick auf intra- und subretinale Flüssigkeit, Membranen und Abhebungshöhen analysiert werden. Möglicherweise beeinflussen die gewonnenen Erkenntnisse zukünftig operative Vorgehensweisen.

## Methoden

Dieser Arbeit liegen die klinischen Daten von 41 Augen von 41 konsekutiven Patienten zugrunde, die im Zeitraum von 6 Monaten in einer Universitäts-Augenklinik mit Maximalversorgung aufgrund einer präoperativ ophthalmoskopisch diagnostizierten Netzhautablösung mit Makulabeteiligung und unter Verwendung einer iOCT behandelt wurden.

Die Einschlusskriterien waren: (1) Ablatio retinae mit Makulabeteiligung bei Indikationsstellung, (2) Operation durch Pars-plana-Vitrektomie (ppV) mit iOCT-fähigem Mikroskop (Lumera Rescan, Carl Zeiss Meditec AG, Jena), (3) vollständige Patientendaten aus der Krankenakte verfügbar, (4) Alter >18 Jahre, (5) Einblick ausreichend für die Generierung eines iOCT-Bildes und (6) ein Operateur (CM). Es wurden keine expliziten Ausschlusskriterien definiert, insbesondere wurden sowohl primäre Eingriffe wie auch Revisionen zugelassen.

Folgende Informationen wurden aus den Patientendokumentationen erhoben: demografische Daten (Alter, Geschlecht), Visus prä- und postoperativ, Symptomdauer, Art und Erweiterungen des operativen Eingriffs (z. B. Makula-Peeling, Tamponade). Die postoperative SD-OCT wurde nach 8 Wochen durchgeführt. Der Dezimalvisus wurde mittels Sehzeichentafel in 5 m oder 1 m Entfernung bestimmt und in logMAR-Werte (engl. „logarithm of the minimum angle of resolution“) umgerechnet.

Alle Patienten erhielten innerhalb von 24 h nach Vorstellung in der Augenklinik eine Standard-3-Port-Vitrektomie in 23-Gauge-Technik. Ein optionales Cerclageband war erlaubt. Sofern sich intraoperativ klinisch im Bereich der Makula ein auffälliger glitzernder Reflex darstellte, wurde die Entscheidung zum ILM-Entfernung getroffen.

Falls ein Membranpeeling erforderlich war, wurde als Farbstoff Brilliant Blue G: 0,125 mg (0,25 g/l; ILM-BLUE® D.O.R.C, Niederlande) verwendet. Während der Operation wurde bei allen Eingriffen schwere Flüssigkeit (Perfluorodecalin [PFCL], Fluoron, Ulm, Deutschland) angewendet, um eine Vergleichbarkeit der Befunde zu gewährleisten. Als Endotamponade wurde entweder steriles Gas (SF6, C2F6 oder C3F8; EasyGas, Fluoron, Ulm, Deutschland) oder Silikonöl (Siluron 2000, Fluoron, Ulm, Deutschland) verwendet. Eine kombinierte ppV mit Phakoemulsifikation und Linsenimplantation wurde durchgeführt, falls dies nötig war.

**Figure 1:**
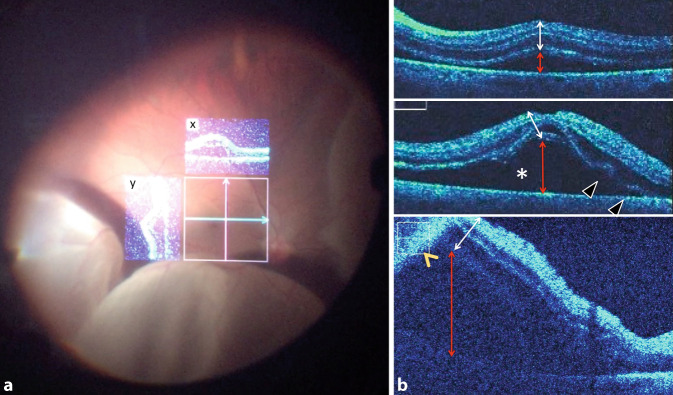

**Figure 2:**
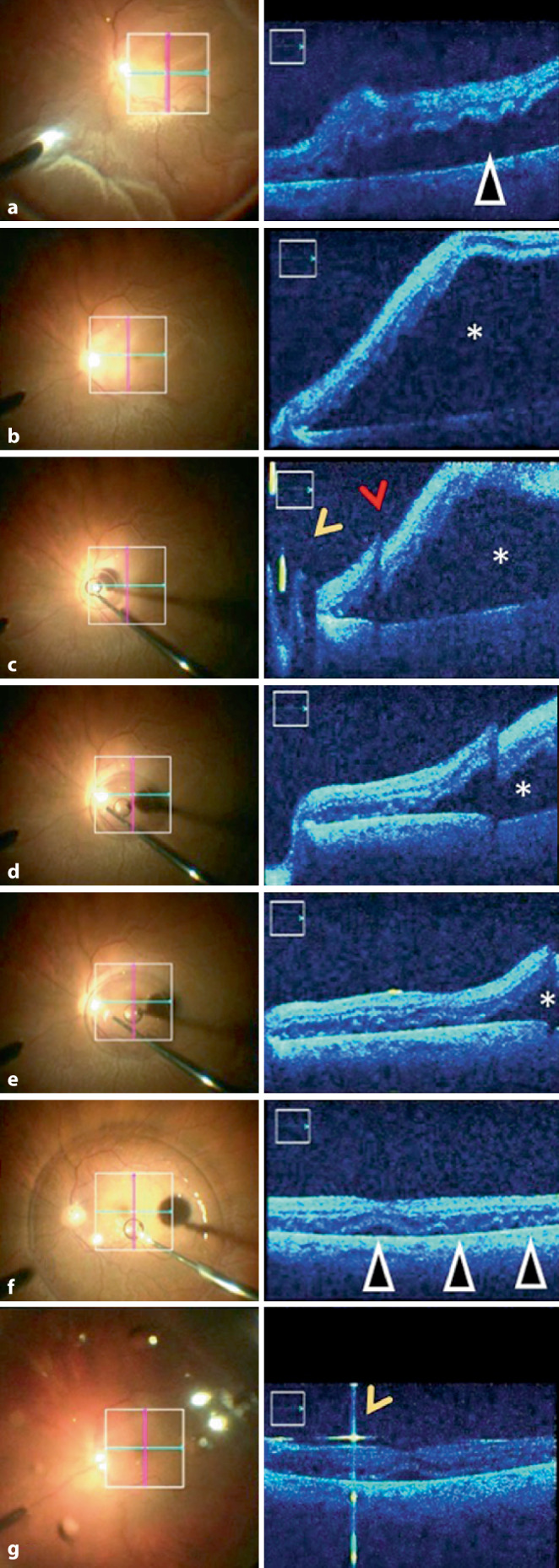

Das Bild des Operationsmikroskops (En-Face und iOCT) wurde digital als Video zur späteren Analyse aufgezeichnet. Die Abb. 1a zeigt die Ansicht durch das Okular des Operationsmikroskops am Beispiel einer hochbullösen Ablatio retinae, wie sie der Operateur sieht. Die Schnittbilder der x‑ und y‑Achse sind im En-face-Bild eingeblendet. Die intraoperative Beurteilung der zentralen Netzhaut mittels iOCT erfolgte zu 3 Zeitpunkten:

1. Zunächst wurde unmittelbar nach Eingehen mit dem Endolichtleiter und vor Beginn jeglicher Manipulation im Glaskörper die Höhe der Netzhautabhebung direkt unterhalb der Fovea bestimmt (Abb. 1b, roter Doppelpfeil), welche anhand der Netzhautdicke in kleiner (a) oder größer (b) einer Netzhautdicke (Abb. 1b, jeweils weißer Doppelpfeil) bzw. höher als der iOCT-Scan (c) quantifiziert wurde (Abb. 1b und 2a).
2. Nach durchgeführter Vitrektomie und nach Eingabe der schweren Flüssigkeit (PFCL) erfolgte die erneute Beurteilung hinsichtlich Netzhautanlage und Morphologie (intra-/subretinale Flüssigkeit) der zentralen Makula (Abb. 2b–f).
3. Nach den gleichen Kriterien erfolgte die abschließende Beurteilung am Ende des Eingriffs nach Injektion der endgültigen Tamponade und vor dem Entfernen der intraokularen Instrumente und Zugänge (Abb. 2g).

Anhand der Operationsvideos wurden retrospektiv auch weitere Besonderheiten des Netzhautverhaltens dokumentiert, wie beispielsweise die Falten der äußeren Netzhautschichten (Abb. 1b, Pfeilspitzen).

Ziel der Arbeit war die Evaluation von iOCT-Makula-Bildern zu Beginn, während und unmittelbar am Ende der Operation. Die demografischen und klinischen Charakteristika wurden unter Verwendung traditioneller deskriptiver Methoden retrospektiv ausgewertet und im Einklang mit nationalem Recht sowie in Übereinstimmung mit der Deklaration von Helsinki von 1975 (in ihrer aktuellen, überarbeiteten Fassung) durchgeführt.

## Ergebnisse

Die Analyse umfasste 41 Patienten mit einem medianen Alter von 61 Jahren. Bei allen Patienten wurde eine klinisch und in der OCT gesicherte Netzhautablösung mit Foveabeteiligung festgestellt. Die Tab. 1 zeigt die Baseline-Charakteristika. Präoperativ lag die mittlere bestkorrigierte Sehschärfe bei 1,6 ± 0,7 logMAR; 14 von 41 (34,1 %) Augen hatten bereits eine operativ versorgte Netzhautablösung in der Vorgeschichte. Im Folgenden sind die Befunde während der 3 Untersuchungsabschnitte dargestellt.

### 1. iOCT-Makulabefund unmittelbar nach Setzen der Trokare, vor Beginn der Vitrektomie (Abb. 2a und 3a, e, i, m, q):

Zu Beginn der Operation ohne jegliche Glaskörpermanipulation zeigte sich bei 12 von 41 (29,3 %) der Patienten eine Elevation von weniger als einer Netzhautdicke, bei 11 von 41 Patienten (26,8 %) eine Elevation von mehr als einer Netzhautdicke (Abb. 1b mittleres Bild, 2g, 2j), sowie bei 15 von 41 Patienten (36,6 %) eine Elevation von einer Abhebung, die höher war, als die Scantiefe zuließ.

**Table 1:** 

Alter in Jahren (Median, SD)	61 ± 13
Geschlecht: männlich/weiblich (*n*)	32/9
Dauer der Symptome bis zur OP (Tage, SD)	26 ± 60
Lateralität: rechtes Auge/linkes Auge (*n*)	23/18
Präoperativer BCVA am betroffenen Auge (logMAR, SD)	1,6 ± 0,7
Re-Ablatio retinae	14/41 (34,1 %)
**Tamponaden**	
SF6 (*n*)	3 (7 %)
C2F6 (*n*)	15 (37 %)
C3F8 (*n*)	1 (2 %)
Silikonöl 2000c (*n*)	22 (54 %)

**Figure 3:**
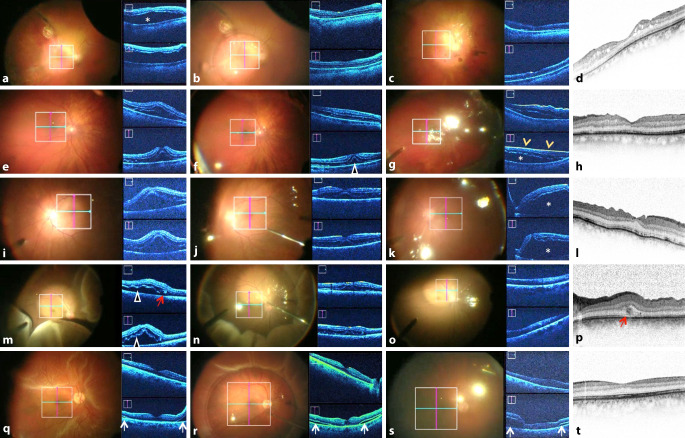

**Figure 4:**
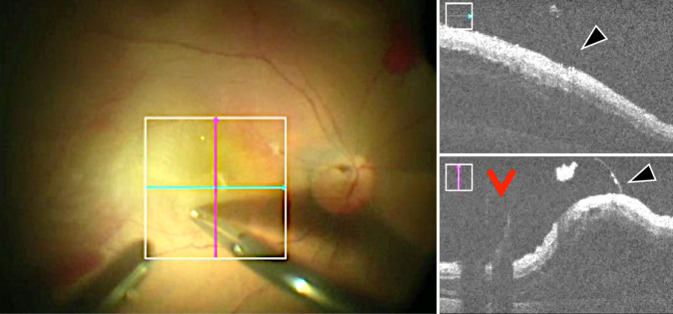

In 3 Fällen (7,3 %) zeigte sich wider Erwarten eine Ablatio mit anliegender Makula (Abb. 3q). Aufgrund eines intraoperativ auffälligen glitzernden Reflexes im Bereich der Makula wurde in 14 Fällen (34,1 %) zusätzlich eine ILM-Entfernung durchgeführt (Abb. 4). Eine epiretinale Membran konnte in diesen Fällen nicht mittels iOCT dargestellt werden. Bei 15 Patienten (36,6 %) konnte eine epiretinale PVR-Reaktion in der iOCT dargestellt werden, die im Folgenden operativ entfernt wurde. In einem Fall zeigte sich ein durchgreifendes Makulaforamen, das mit Membranpeeling unter Ablatiobedingungen und Gaseingabe behandelt wurde. Somit erfolgte insgesamt bei 30 von 41 Patienten (73,2 %) mit Ablatio retinae und Makulabeteiligung eine Membranentfernung.

### 2. iOCT-Makulabefund unter Perfluorodecalin (Abb. 2b–f und 3b, f, j, n, r):

Unter PFCL lag die makuläre Netzhaut vollständig bei 15 von 41 Patienten (36,6 %) an, während bei 14 Patienten (34,1 %) noch ein dünner subretinaler Flüssigkeitsfilm nachgewiesen werden konnte, welcher sich offenbar nicht vollständig durch die schwere Flüssigkeit verdrängen ließ (Abb. 2b–f). Bei 4 Patienten (9,8 %) zeigten sich unter PFCL intraretinale Zysten. Bei 8 (19 %) Patienten konnten sowohl intraretinale Zysten als auch subretinale Flüssigkeit unter PFCL nachgewiesen werden. Zusätzlich konnten in 22 % der Fälle (9/41 Augen) Falten der äußeren Netzhautschichten beobachtet werden (Abb. 2b–f und 3e). Ein Fall einer Ablatio retinae mit anliegender Fovea (Abb. 3r) zeigte unter PFCL parafoveal noch einen schmalen subretinalen Flüssigkeitsfilm (Abb. 3s, weiße Pfeile). PFCL ließ sich in der iOCT nicht darstellen.

### 3. iOCT-Makulabefund unter der endgültigen Endotamponade vor Entfernen der Trokare (Abb. 2g und 3c, g, k, o, s):

Nach Eingabe der endgültigen Endotamponade wurde eine abschließende Beurteilung der Netzhaut mittels iOCT vorgenommen. Die Abb. 2b–g zeigt exemplarisch eine anliegende Netzhaut unter Gastamponade mit gut sichtbarer Gas-Flüssigkeits-Grenze sowie persistierender submakulärer Flüssigkeit sowie Falten der äußeren Netzhautschichten. Bei 14 Patienten (39 %) konnte eine sofortige, vollständige Netzhautanlage direkt am Ende der Operation nachgewiesen werden, bei 25 Patienten (60,9 %) fand sich weiterhin persistierende oder aus der Peripherie nach zentral verlagerte subretinale Flüssigkeit in unterschiedlichem Ausmaß. Unabhängig von der Netzhautanlage unter PFCL konnte bei 7 Patienten (17,1 %) intraretinale Flüssigkeit bzw. eine Netzhautverdickung festgestellt werden.

Als Endotamponade wurde in 19 Fällen Gas (SF6, C2F6 oder C3F8) und in 22 Fällen Silikonöl verwendet.

Die Tab. 2 zeigt zusammengefasst die intraoperativ erhobenen Netzhautbefunde. Am Entlasstag lag die bestkorrigierte Sehschärfe bei 2,0 ± 0,5 logMAR. Acht Wochen nach dem chirurgischen Eingriff erfolgte eine Follow-up-Untersuchung: Die bestkorrigierte Sehschärfe betrug 0,85 ± 0,7 logMAR. In der zu diesem Zeitpunkt erneuten OCT-Untersuchung zeigte sich in 5 % der Fälle noch subretinale Flüssigkeit im Makulabereich (Abb. 3g, k), in 38 % der Fälle noch intraretinale Flüssigkeit, während keine sichtbaren makulären Gliosen oder epiretinale Membranen gefunden wurden. In der Hälfte der Fälle wurden andere morphologische Netzhautveränderungen festgestellt: verdickte Netzhaut (*n* = 3), Makulaschichtforamina (*n* = 2), verstrichene Foveakonturen (*n* = 2), Verdacht auf chorioidale Neovaskularisation (*n* = 1), subretinaler Strang (*n* = 1, Abb. 3p, roter Pfeil), Falten der Ganglienzellschicht (*n* = 1, Abb. 3l), verdicktes retinales Pigmentepithel (*n* = 1), Makulaödem (*n* = 1 Fall). Im Nachbeobachtungszeitraum zeigte sich bei keinem Fall eine Reablatio.

**Table 2:** 

	*n* (%)
**Netzhautbefund vor Beginn der eigentlichen Vitrektomie**	
Abhebung weniger als eine Netzhautdicke	12 (29,3)
Abhebung mehr als eine Netzhautdicke	11 (26,8)
Abhebung größer als maximale Scantiefe	15 (36,6)
Anliegende Makula	3 (7,3)
**Netzhautbefund nach Vitrektomie und PFCL-Eingabe**	
Sofortige, vollständige Netzhautanlage	15 (36,6)
Persistierende subretinale Flüssigkeit	14 (34,1)
Intraretinale Flüssigkeit	4 (9,8)
Intra- und subretinale Flüssigkeit	8 (19,5)
**Netzhautbefund unter endgültiger Tamponade**	
Sofortige, vollständige Netzhautanlage	14 (39)
Persistierende subretinale Flüssigkeit	25 (60,9)
Intraretinale Flüssigkeit	7 (17,1)
**Allgemeine Befunde während der Operation**	
Epiretinale Gliose	14 (34,1)
Epiretinale PVR-Reaktion	15 (36,6)
Makulaforamen	1 (2,4)
Falten der äußeren Netzhautschichten	9 (22)

## Diskussion

Die intraoperative OCT bietet die Möglichkeit, in Echtzeit Feinschichtaufnahmen der Netzhaut des hinteren Pols während einer Vitrektomie durchzuführen. Leiser et al. konnten zeigen, dass die intraoperative Spectral-Domain-OCT eine hohe Reproduzierbarkeit hinsichtlich der Sichtbarkeit von epiretinalen Membranen (ERM), lamellären Makulalöchern und vitreomakulärer Traktion bietet [12]. Allerdings verursachten mikrozystische Veränderungen Diskrepanzen bei der Interpretation, die oft einfach als Netzhautverdickung diagnostiziert wurden. In einer anderen Arbeit berichtete dieselbe Arbeitsgruppe darüber [13], dass dank der iOCT ein ERM-Peeling ohne Färbung in der Mehrheit der Fälle (63 %) durchgeführt werden konnte. Auch wir sehen die Echtzeitvisualisierung des Netzhautverhaltens unter Manipulation und damit die direkte Rückmeldung des In-situ-Befundes als besonders hilfreich an. Die vorliegende Arbeit untersucht die möglichen Mehrgewinne der Diagnostik anhand von makulabeteiligten Netzhautablösungen. In 63 % der hier untersuchten Fälle gelang die iOCT-Darstellung der Makula zu Beginn der Operation. In den anderen Fällen überstieg die Höhe der Netzhautablösung die maximale Scanhöhe der iOCT. Unter Ablatiobedingungen scheint daher die Darstellung mittels iOCT in den meisten Fällen möglich, jedoch ist insbesondere bei hochblasigen Ablationes die Verwendbarkeit der iOCT zumindest zu Beginn der Operation wenig aussagekräftig. Die Höhe der initialen fovealen Abhebung konnte nicht in Zusammenhang mit anderen Befunden während und am Ende der Operation gebracht werden. Abraham et al. [1] konnten allerdings zeigen, dass Netzhautchirurgen in komplexen Amotiones die durch die iOCT vermittelten, zusätzlichen Informationen signifikant häufiger als hilfreich empfanden als in einfachen Fällen. Auch in unserer Arbeit zeigten sich zusätzliche Informationen, die zuvor in dieser Weise nicht ersichtlich waren:

Trotz präoperativ klinisch diagnostizierter Makula-off-Ablatio konnte intraoperativ in 3 Fällen eine anliegende Fovea darstellt werden. Möglicherweise handelt es sich in diesen Fällen um eine Spontananlage, welche durch die Lagerung (prä- und intraoperativ) bedingt war. Auch in dieser Studie konnten wir ein zuvor klinisch nicht diagnostiziertes Makulaforamen detektieren. Ehlers et al. [7] berichteten ebenfalls darüber, intraoperativ durch die Verwendung einer iOCT ein zuvor nicht erkanntes Makulaforamen diagnostiziert zu haben. Dieses Phänomen lässt sich möglicherweise dadurch erklären, dass präoperativ entweder keine SD-OCT durchgeführt wurde und somit vermutlich klinisch übersehen wurde oder das Foramen nicht vom SD-OCT-Scan getroffen wurde.

Überraschenderweise ließ sich mittels der iOCT in über 60 % noch persistierende subretinale Flüssigkeit am Ende der OP nachweisen, wobei dies nicht der visuellen klinischen Einschätzung durch das Mikroskop entsprach. Es ist unklar, ob der dünne persistierende subretinale Flüssigkeitsfilm die Ursache für die beobachteten Falten der äußeren Netzhautschichten ist oder umgekehrt. Ehlers et al. [7] berichten ebenso von subretinaler Flüssigkeit und Falten der äußeren Netzhautschichten in 100 % der Fälle (9/9 Augen) nach Injektion von Perfluorooctan (PFO). Junker et al. [10] konnten diese Ergebnisse zumindest teilweise bestätigen. In unserer Studie konnten persistierende subretinale Flüssigkeit in 60,9 % der Fälle (25/41 Augen) und Falten der äußeren Netzhautschichten in 22 % der Fälle (9/41 Augen) nach Injektion von PFCL festgestellt werden. Junker et al. [10] vermuten, dass die subretinale Flüssigkeit oder Falten der äußeren Netzhautschichten durch die unterschiedlichen Dichten der schweren Flüssigkeit verursacht sein könnten. Cho et al. [4] konnten zeigen, dass präoperativ beobachtete Falten der äußeren Netzhautschichten ein negativer prädiktiver Faktor sind. Wir konnten zeigen, dass diese Veränderungen auch intraoperativ und zum Teil noch am Ende der Operation darstellbar sind. Über die Zeit, die der Eingriff dauerte, und damit der Anpressdruck der schweren Flüssigkeit wirken konnte, schien die Menge der subretinalen Flüssigkeit abzunehmen (Abb. 2). Die klinische Relevanz von persistierender subretinaler Flüssigkeit am Ende der Operation ist derzeit noch unklar. Die Menge der subretinalen Flüssigkeit scheint unterschiedlich groß zu sein und ist möglicherweise durch die Zusammensetzung und das Vorhandensein von Reservoirs (i. S. des „Fluid-Shifting“) bedingt. Die Beobachtung, dass die Makula häufiger unter PFCL als unter der endgültigen Endotamponade anliegt, spricht für dieses Phänomen. Das retinale Pigmentepithel resorbierte im Verlauf alle noch vorhandene subretinale Flüssigkeit. Ob die entfernte Menge zu einem unterschiedlichen postoperativen Visus führt, ist anhand unserer Daten nicht zu eruieren. Hierzu wäre eine Studie mit Quantifizierung der subretinalen Flüssigkeit wünschenswert. Zudem besteht unmittelbar postoperativ ein diagnostisches blindes Fenster (v. a. im Falle einer Gastamponade) für OCT-Aufnahmen. Inwiefern die postoperativen OCT-Aufnahmen 8 Wochen später in Relation zu den intraoperativen Befunden stehen, lässt sich in dieser Analyse nicht herausarbeiten. Die intra- und auch die postoperativen Befunde waren zu heterogen (Dauer der Ablatio, Ursache, Ausprägung und Art der Versorgung), als dass ein aussagekräftiger Zusammenhang ausgewertet werden konnte. Zusätzlich ist die Aussagekraft durch intraoperative iOCT-Bildqualität im Vergleich zu der höher auflösenden Darstellung in den standardmäßig gemittelten Aufnahmebildern der postoperativen SD-OCT reduziert. Für eine solche Fragestellung bedarf es eines anderen Studienaufbaus.

Während die Endotamponaden (Gas und Silikonöl) an ihrem Spiegel gut darstellbar waren (s. Abb. 2g), konnte die schwere Flüssigkeitsgrenze (PFCL) nicht dargestellt werden. Damit scheint die iOCT eher ungeeignet dafür zu sein, die vollständige Entfernung von PFCL zu überwachen. Subretinales PFCL konnten wir in unserer Kohorte (glücklicherweise) nicht darstellen, wäre aber in der regulären OCT gut darstellbar. In 54 % der Fälle (22/41 Augen) wurde Silikonöl als Endotamponade gewählt. Dieser hohe Anteil ist am ehesten dadurch bedingt, dass auch 14 von 41 Augen mit Re-Ablationes sowie weitere mit komplexen Ausgangsituationen in die Studie eingeschlossen wurden. Außerdem stellen die Netzhautablösungen mit Makulabeteiligung ein tendenziell weiter fortgeschrittenes Krankheitsbild dar.

Unsere Arbeit hat einige Limitationen. Angesichts des heterogenen Patientenkollektivs, der geringen Fallzahl sowie der fehlenden Kontrollgruppe können Fragen zum Einfluss der SRF oder des Peelings auf den postoperativen Visus nicht ausreichend beantwortet werden.

Einige Autoren sehen keine offensichtlichen Vorteile beim Einsatz der iOCT bei der Netzhautablösung. Dennoch bietet diese neue Technik tiefere Einblicke in die Mikroarchitektur der abgelösten Netzhaut [10]. Im Wesentlichen beeinflusst die iOCT weder die Handhabung des Operationsmikroskops noch den Routineablauf der Ablatio-OP. In einigen Fällen konnte durch die iOCT der klinische Eindruck der Netzhautsituation korrigiert werden, etwa durch die Darstellung persistierender subretinaler Flüssigkeit bei klinischem Eindruck einer ausreichenden „Trockenlegung“ der Netzhaut. Dieses Ergebnis ähnelt dem von Abraham et al. [1], welcher über eine iOCT-getriggerte Änderung seiner chirurgischen Strategie in 12 % der Fälle beschreibt. Weitere Untersuchungen bei mehr Patienten werden zeigen, ob der Einsatz der iOCT zu einer besseren Prognose für unsere Patienten führt.

Zusammenfassend führen die Informationen durch die iOCT in der Ablatiochirurgie bisher eher selten zu einer Änderung oder Erweiterung des Eingriffs. Sie liefert jedoch in Echtzeit neue Informationen wie intra- und subretinale Flüssigkeitsverteilungen v. a. in Querschnittaufnahmen der Netzhaut, die im Operationsmikroskop nur in der Aufsicht zu erfassen sind. Damit führt die iOCT zu einem besseren Verständnis über das Verhalten der Netzhaut direkt und unmittelbar unter laufenden OP-Bedingungen.

## Fazit für die Praxis

- Die iOCT erlaubt während des Eingriffs die Echtzeitdarstellung ansonsten nicht sichtbarer retinaler Strukturen direkt im Operationsmikroskop und können dadurch evtl. unmittelbar behandelt werden.
- In der Netzhautchirurgie bei Amotio retinae mit Makulabeteiligung zeigt sich oft subfoveale Flüssigkeit, die nicht immer dem intraoperativen klinischen Eindruck entspricht.
- Die Bedeutung von intra-, subretinaler Flüssigkeit und Falten der äußeren Netzhautschichten am Ende der OP ist derzeit noch unklar, sie könnten jedoch zukünftig als prognostische Faktoren für das postoperative Outcome dienen.
